# The role of point-of-care ultrasound in the diagnosis of pericardial effusion: a single academic center retrospective study

**DOI:** 10.1186/s13089-021-00205-x

**Published:** 2021-02-04

**Authors:** Matthew G. Hanson, Barry Chan

**Affiliations:** 1 Division of Cardiology, Department of Medicine, Queen’s University, Kingston, ON Canada; 2 Division of General Internal Medicine, Department of Medicine, Queen’s University, Kingston, ON Canada

**Keywords:** Pericardial effusion, Point-of-care ultrasound, Diagnosis

## Abstract

**Background:**

Symptomatic pericardial effusion (PCE) presents with non-specific features and are often missed on the initial physical exam, chest X-ray (CXR), and electrocardiogram (ECG). In extreme cases, misdiagnosis can evolve into decompensated cardiac tamponade, a life-threatening obstructive shock. The purpose of this study is to evaluate the impact of point-of-care ultrasound (POCUS) on the diagnosis and therapeutic intervention of clinically significant PCE.

**Methods:**

In a retrospective chart review, we looked at all patients between 2002 and 2018 at a major Canadian academic hospital who had a pericardiocentesis for clinically significant PCE. We extracted the rate of presenting complaints, physical exam findings, X-ray findings, ECG findings, time-to-diagnosis, and time-to-pericardiocentesis and how these were impacted by POCUS.

**Results:**

The most common presenting symptom was dyspnea (64%) and the average systolic blood pressure (SBP) was 120 mmHg. 86% of people presenting had an effusion > 1 cm, and 89% were circumferential on departmental echocardiogram (ECHO) with 64% having evidence of right atrial systolic collapse and 58% with early diastolic right ventricular collapse. The average time-to-diagnosis with POCUS was 5.9 h compared to > 12 h with other imaging including departmental ECHO. Those who had the PCE identified by POCUS had an average time-to-pericardiocentesis of 28.1 h compared to > 48 h with other diagnostic modalities.

**Conclusion:**

POCUS expedites the diagnosis of symptomatic PCE given its non-specific clinical findings which, in turn, may accelerate the time-to-intervention.

## Introduction

Pericardial effusion (PCE) is a fluid collection in the pericardial sac which can result in life-threatening cardiac tamponade. Cardiac tamponade occurs when the intrapericardial pressure compresses the cardiac chambers resulting in impaired diastolic filling for which the right heart is most susceptible [1, 2]. The fibrous pericardial sac has limited compliance; therefore, tamponade can be precipitated when small volume of fluid accumulates quickly [3, 4]. Alternatively, subacute accumulation can stretch the pericardium, increasing its compliance, such that higher PCE volume is required to result in tamponade [1, 2, 5]. The pericardial sac generally contains 50 mL of fluid, and effusion size is classified based on the pericardial separation distance during diastole with small effusion being < 10 mm (50–100 mL), moderate effusion as 10–20 mm (100–500 mL), and large effusion > 20 mm (> 500 mL) [4, 6, 7].

Often patients with symptomatic PCE or tamponade present with non-specific symptoms and signs such as dyspnea, peripheral edema, tachycardia, and elevated jugular venous pressure (JVP) [2, 5]. As such, bedside diagnosis is challenging given the non-specific symptomatology and variable exam findings [3, 5]. Classically, Beck’s Triad, comprising hypotension, increased JVP, and quiet/distant heart sounds, has been taught to screen for tamponade at the bedside [2, 8, 9]. However, the triad does not reliably predict the presence of tamponade or significant PCE in over 40% of cases [5]. Other clinical features classically described for PCE or tamponade include tachypnea, tachycardia, and pulsus paradoxus are non-specific [2]. For example, pulsus paradoxus may be present in patients with significant respiratory distress (asthma or chronic obstructive pulmonary disease exacerbation), or any mechanism that exaggerates the interventricular dependence. Conversely, pulsus paradoxus can be absent in patients with left ventricular hypertrophy, hypovolemia or atrial septal defects even in the presence of clinically significant PCE [2, 10, 11]. Some studies have looked at adjuncts to the physical exam including electrocardiogram (ECG) analysis, using chest X-ray (CXR), or point-of-care ultrasound (POCUS) to aid the diagnosis of PCE or cardiac tamponade [5, 8, 10, 12–14]. ECG and CXR lack diagnostic sensitivity, whereas POCUS has been shown to be sensitive, specific, and potentially reduces the time-to-intervention [1, 10, 12].

Echocardiography (ECHO) remains as the gold standard imaging modality to verify the presence of a PCE by demonstrating fluid collection in the pericardial space and interrogating for features of tamponade physiology such as systolic right atrial (RA) collapse, early diastolic right ventricle (RV) collapse, respiratory variations of transvalvular flow [1, 3, 4, 7].

To determine the rate of reported symptoms, physical exam findings, ECG and CXR abnormalities, we looked at all patients who underwent pericardiocentesis between 2002 and 2018 at a Canadian tertiary care hospital. Furthermore, we looked at ECHO findings, the length of time-to-diagnosis and time-to-pericardiocentesis, and how these were affected by POCUS.

## Methods

### Study design

This chart review was performed from 2002–2018 on patients who underwent pericardiocentesis at Kingston Health Sciences Centre, a tertiary care hospital in Kingston, Ontario, Canada. Ethical consent was obtained from the Queen’s University Health Sciences & Affiliated Teaching Hospitals Research Ethics Board. Patient electronic medical records were searched for the terms “pericardiocentesis + pericardial tamponade”, “pericardiocentesis + cardiac tamponade”, and “pericardiocentesis + pericardial effusion”.

Patient records were reviewed for the presenting symptomatology, physical examination findings, CXR and ECG reports, POCUS use, time-to-diagnosis, and time-to-pericardiocentesis where available. Demographic data including age, gender, past medical history, and medications were, also, obtained where available.

In terms of POCUS resource at our institution, the prevalence of trained POCUS users in 2002 is unknown though its use was limited to Emergency Medicine. By 2018, 100% of the Emergency Physicians were POCUS users with the majority trained via the Emergency Department ECHO (EDE) 1 program; and 30% of General Internal Medicine were POCUS users with training ranging from part of their subspecialty fellowship program to proprietary training courses such as the Canadian Point of Care Ultrasound Society (CPOCUS). With regard to POCUS machinery, the first mention of the use of POCUS in our study was 2007, at the time, there was a total of 3 POCUS machines all of which were situated in the emergency room. As of 2018, there were 9 machines (1 for Cardiology, 2 for Critical Care, 3 for Emergency Medicine, 3 for General Internal Medicine).

As there is no POCUS archiving and reporting system locally, the beside sonographic findings and interpretation were extracted from written documentation (paper chart or electronic medical record). The CXR and ECG interpretations were derived from the finalized reports of the respective radiologist and cardiologist.

The time-to-diagnosis interval was computed from the time of presentation to the earliest mention of the presence of a PCE for a respective diagnostic modality. The time-to-pericardiocentesis interval was computed from the time of presentation to the earliest mention of the procedure being commenced or underway. Patients requiring more than one pericardiocentesis during a given visit only had the first procedure considered for the study. The differences in the means of the presenting hemodynamic profiles between the groups that was first diagnosed via POCUS and departmental ECHO were analyzed with the Welch’s *t*-test with the threshold p-value adjusted through the Bonferroni correction.

Data are reported as an overall average of all charts and include those where data were unavailable in the denominator unless otherwise specified. Statistical analysis was performed with Statistical Package for Social Sciences (SPSS) Version 26.

## Results

### Baseline characteristics

#### The search algorithm returned 342 unique charts

In all comers receiving pericardiocentesis, the average age was 63.3 ± 14 years and males were slightly more common than females (56 vs 44%, respectively). The most common comorbid conditions were hypertension (37%) and malignancy (27%) (Table 1). 79 were procedurally related PCE (23%) with ablation for atrial fibrillation/flutter being the most common cause.

**Table 1:** Baseline characteristics (*n* = 342)

Age (years)	63.3 ± 14.1
Gender	*n* (%)
Male	193 (56)
Female	149 (44)
Cardiovascular risk factors	
Hypertension	127 (37)
Diabetes	65 (19)
Coronary artery disease	59 (17)
Atrial fibrillation/flutter	63 (18)
Inflammatory conditions	
Rheumatologic conditions	25 (7)
Pericarditis	10 (3)
Malignancy	91 (27)
Recent cardiac procedure	31 (9)
Hemodynamics on presentation	
Systolic blood pressure (mmHg)	121 ± 27
Diastolic blood pressure (mmHg)	72 ± 14
Systolic blood pressure ≤ 90 mmHg (*n*) (%)	33 (9.6)
Heart rate (beats per minute)	94 ± 22
On oral anticoagulation	
Warfarin	26 (8)
Direct oral anticoagulant	5 (1)
Other cardiac medications	
ACE inhibitor/angiotensin receptor blocker	88 (26)
Alpha-blocker	18 (5)
Beta-blocker	77 (23)
Dihydropyridine calcium channel blocker	37 (11)
Non-dihydropyridine calcium channel blocker	22 (6)
Diuretics	81 (24)
Anti-arrhythmics	29 (8)
Vasodilators	9 (3)

Upon review of all the charts available via the search terms which included “Pericardiocentesis”, the procedures were all performed as inpatient.

### Most common presentation complaint, physical exam, ECG, CXR findings

The most common presenting complaint was dyspnea which was present in 66% of patients; and 41% had elevated JVP.

Most patients had compensated initial hemodynamics where 43% had a normal heart rate and only 9.6% were hypotensive with SBP ≤ 90 mmHg.

47% had tachycardia on ECG at the time of presentation; and 54% had cardiomegaly on CXR. The remainder of other clinical features, ECG, and radiographic findings are tabulated in Table 2.

**Table 2:** Presenting symptoms, physical exam, electrocardiogram, and chest X-ray findings (*n* = 342)

	Yes *n* (%)	No *n* (%)	Not available *n* (%)
Presenting symptoms			
Dyspnea	227 (66)	52 (15)	63 (18)
Orthopnea	51 (15)	151 (44)	140 (41)
Chest pain	108 (32)	151 (44)	83 (24)
Presyncope	45 (13)	184 (54)	113 (33)
Syncope	20 (6)	208 (61)	114 (33)
Peripheral edema	41 (12)	189 (55)	112 (33)
Physical exam			
Respiratory distress	67 (20)	183 (54)	220 (64)
Pulsus paradoxus (> 10 mmHg)	123 (36)	168 (49)	51 (15)
Muffled heart sound	10 (3)	271 (79)	61 (18)
Elevated jugular venous pulse	140 (41)	188 (55)	14 (4)
Electrocardiogram			
Sinus tachycardia	161 (47)	153 (45)	28 (8)
Low voltage	123 (36)	168 (49)	51 (15)
Alternans	10 (3)	271 (79)	61 (18)
Chest X-ray			
Cardiomegaly	184 (54)	55 (16)	103 (30)
Pulmonary edema	15 (4)	227 (66)	100 (29)

### Modality of initial diagnosis, time-to-diagnosis, and time-to-intervention

For non-procedurally related PCE, 35.5% were identified by departmental ECHO, 23.3% via POCUS, and 19.8% through other imaging modalities for which computed tomography (CT) scan was most common.

The time-to-diagnosis with POCUS and departmental ECHO were on average 5.9 h and 45.1 h, respectively. Time-to-pericardiocentesis was 28.1 h in the POCUS group in comparison to 49 h for the departmental ECHO group (Table 3). After adjustment with the Bonferroni correction (*p*-value threshold < 0.0167 is considered to indicate statistical significance), there was no statistical difference between the presenting hemodynamic profile between the two groups (Table 4).

**Table 3:** Diagnostic modality, time-to-diagnosis, and time-to-intervention (non-procedure related *n* = 263)

Initial diagnostic modality (*n*)	Time-to-diagnosis in hours (*n*)	Time-to-pericardiocentesis in hours (*n*)
Departmental ECHO (93)	45.1(35)	49.0 (67)
POCUS (61)	5.9 (47)	28.1 (55)
Other^a^ (53)	12.0 (30)	56.1 (45)
Not available (56)	–	–

^a^Other imaging includes CT (*n* = 49), abdominal ultrasound (*n* = 1), cardiac MRI (*n* = 1), and ventilation/perfusion (VQ) scan (*n* = 1), intraoperative transesophageal ECHO (*n* = 1)

**Table 4:** Comparison of hemodynamics on presentation of the POCUS and departmental ECHO groups with the Welch’s *t*-test

	POCUS (*n* = 75)	Departmental ECHO (*n* = 126)	Mean difference	*p*-value^a^
Mean systolic blood pressure (mmHg)	112 (95% CI 107–117)	121 (95% CI 116–126)	8.3 (95% CI 1.2–15.5)	0.022
Mean diastolic blood pressure (mmHg)	73 (95% CI 69–77)	73 95% CI 70–76)	0.63 (95% CI − 3.6–4.9)	0.77
Mean heart rate (beats per minute)	99 (95% CI 94–104)	94 (95% CI 82–106)	− 5.4 (95% CI − 11–0.3)	0.63

^a^With the Bonferroni adjustment, a *p*-value of < 0.0167 is considered to indicate statistical significance

### Echocardiography

Of all patients who received a pericardiocentesis and had a measured effusion pocket, 67% had a PCE ≥ 2 cm, 17% were 1–2 cm, and 3.5% with < 1 cm. 89% had circumferential effusion (Table 5). 64% had evidence of systolic right atrial (RA) collapse, and 58% had early diastolic right ventricular (RV) collapse.

**Table 5:** Departmental echocardiographic findings

	All (*N* = 342)			Non-procedural etiology (*N* = 263)			Iatrogenic/procedural etiology (*N* = 79)		
	Yes	No	Not available	Yes	No	Not available	Yes	No	Not available
Right atrial collapse	219 (64%)	68 (20%)	55 (16%)	187 (71%)	47 (18%)	29 (11%)	32 (41%)	21 (27%)	26 (33%)
Right ventricle collapse	199 (58%)	89 (26%)	54 (16%)	164 (62%)	7 (27%)	28 (11%)	35 (44%)	18 (23%)	26 (33%)
Left atrial collapse	10 (3%)	275 (80%)	57 (17%)	8 (3%)	225 (86%)	30 (11%)	2 (3%)	50 (63%)	27 (34%)
Left ventricle collapse	4 (1%)	282 (83%)	56 (16%)	2 (1%)	231 (89%)	30 (11%)	2 (3%)	51 (65%)	26 (33%)
Significant respiratory variation	98 (29%)	41 (12%)	203 (59%)	86 (33%)	33 (13%)	144 (55%)	12 (15%)	8 (10%)	59 (75%)
Mechanical alternans	8 (2%)	36 (11%)	298 (87%)	8 (3%)	32 (12%)	223 (85%)	0 (0%)	4 (5%)	75 (95%)
Effusion location									
Circumferential	307 (90%)			255 (97%)			52 (66%)		
Regional	19 (6%)			6 (2%)			13 (16%)		
Not available	16 (4%)			2 (1%)			14 (18%)		
Deepest effusion pocket (measured during diastole)									
< 1 cm	13 (4%)			5 (2%)			8 (10%)		
1–2 cm	57 (17%)			41 (16%)			16 (20%)		
> 2 cm	228 (67%)			204 (78%)			24 (30%)		
Not available	33 (10%)			11 (4%)			22 (28%)		
Pericardial clot	11 (3%)			2 (1%)			9 (11%)		

## Discussion

PCE is challenging to diagnose clinically. Larose et al. noted 80% of 50 cardiac tamponade who necessitated pericardiocentesis received either the wrong diagnosis (36%) or not considered as part of the differential diagnosis (44%) [15]. The most common symptom is dyspnea is non-specific for clinically significant PCE. Furthermore, many of the classically taught physical exam findings do not accurately rule in or out PCE. In comparison to a meta-analysis by Jacob et al. to our review, 54% (33–74%) vs 41% had elevated JVP, 22% (11–34%) vs 15% with muffled heart sounds, and hypotension in 28% (14–39%) vs 10%, respectively [5]. The difference in the rates of hypotension could be explained by patient population selection. In the meta-analysis, the study with the highest hypotension rate (39%), iatrogenic PCE accounted for 31% of the cases whereas our study was 23%. Trauma and surgically related PCE tend to develop acute tamponade in comparison to medical etiologies. In addition, majority of the studies took place in the 1980s (1963–1997) and comparing to a more contemporary review [16] only 15% patients presenting to the emergency department with non-traumatic causes of cardiac tamponade were hypotensive on arrival which was similar with our findings. Gandhi et al. also, noted there was less hypotensive presentation (32% vs 15%) comparing cardiac tamponade cohorts from 1998–1991 to 2002–2005 [19]. This could be explained by an increased index of suspicion for the condition over the past decade [19] and access to echocardiography to assist in the diagnosis [20].

In our study, many were hemodynamically compensated on presentation with fewer than 10% being hypotensive despite 84% of all cases had moderate-to-large effusions with > 50% having RA and/or RV collapse. This is in keeping with previous studies that demonstrated normotension or even hypertension in patients with subacute or chronic PCE accumulation resulting in cardiac tamponade [16–18]. These echocardiographic-to-clinical discrepancies illustrate the hemodynamic spectrum spanning from subclinical to clinically overt yet compensated to outright decompensatory shock [11, 18].

Given the insidious nature of PCE, evaluation with POCUS could reduce the time-to-diagnosis which had truncated by 39 h in our study. In terms of time-to-pericardiocentesis, our data demonstrated the POCUS group received the intervention 21 h earlier than the departmental ECHO group. A smaller retrospective study by Alert et al. found a similar signal whereby the POCUS arm received pericardiocentesis 59 h earlier than the control [12]. However, this trend towards diagnostic and interventional efficiency should be interpreted with caution. Intuitively, POCUS would readily allow significant PCE to be identified, therefore leading to earlier diagnosis and intervention if indicated. Conversely, those who are sicker may more likely receive POCUS interrogation by the concerned physician—a selection bias. In our study, however, there was no significant hemodynamic difference between the two groups on presentation. Perhaps this could be explained by an increase use of POCUS as training programs foster the skillset into their curriculums. As of 2012, 100% of Fellow of the Royal College of Physicians of Canada–Emergency Medicine residency programs (FRCPC-EM) and 88% of the Certification in the College of Family Physicians Emergency Medicine (CCFP-EM) residency programs have formally incorporated POCUS as part of their curriculums [21]. A recent national in 2016 amongst Canadian emergency physicians demonstrated an overall increase in POCUS usage compared to 2007 [22]. Critical Care and General Internal Medicine are, also, recommending POCUS training which will foster its clinical utilization [23, 24].

This study has several limitations. First of all, it takes place at a single university hospital, therefore the results may not extrapolate to other systems where the etiological prevalence differs especially in trauma centers.

Secondly, this is a retrospective study spanning from 2002 to 2018 whereby many charts were only partially converted to the electronic version for review and, also, given the lack of systematic history and physical exam findings data documentation, and POCUS archiving system, the dataset is subjected to significant registry bias as certain elements may not have been recorded or explicitly sought. Furthermore, some of the cases were transferred from peripheral hospitals whereby the presenting complaints, vitals, and initial diagnostic tests were not available. Uncaptured PCE that was discovered by POCUS that warranted a pericardiocentesis will affect the time-to-diagnosis and time-to-pericardiocentesis aggregate estimation as well. Nevertheless, as discussed above, the rates determined from our limited study are congruent with to a large meta-analysis [5]. Also, the POCUS group’s time-to-diagnosis was on average 39 h less than the group diagnosed via departmental echocardiography. This time margin is quite wide unlikely explainable by exclusion of undocumented POCUS alone.

This study did not include two groups: (a) those who died from cardiac tamponade as they would not have received pericardiocentesis and (b) those who were diagnosed with a PCE and discharged without a pericardiocentesis. As such, their respective time-to-diagnosis intervals are not incorporated into the calculation. However, given both groups did not receive pericardiocentesis, its exclusion will not affect the time-to-pericardiocentesis estimation. Also, the intent of the study is to investigate only those with clinically significant PCE who warranted pericardiocentesis on the same admission.

The estimation of the time-to-diagnosis and time-to-pericardiocentesis is dependent on timestamps from the time of initial presentation to the date and time when the procedure being performed is recorded. As such, the time frame will not be precise to the minute. Consequently, the interval is reported in hours instead which will be less error prone. However, a prospective study is necessitated to verify the value of POCUS upon these definitive endpoints and with current POCUS archiving systems the analysis can be more rigorous.

## Conclusion

Clinically significant PCE presents with non-specific findings whereby a high degree of clinical suspicion is necessitated. However, POCUS is a readily available bedside diagnostic tool that can quickly rule in the diagnosis. Whether it may expedite definitive intervention, further studies would be required.

## Data Availability

The data compilation is available via the corresponding author.

## Abbreviations

DBP: Diastolic blood pressure
ECHO: Echocardiogram
ECG: Electrocardiogram
CXR: Chest X-ray
JVP: Jugular venous pulse
LA: Left atrium
LV: Left ventricle
PCE: Pericardial effusion
POCUS: Point-of-care ultrasound
RA: Right atrium
RV: Right ventricle
SBP: Systolic blood pressure
VQ: Ventilation perfusion
